# Can the International Conference on Population and Development Programme of Action and Cairo Consensus Normalize the Discourse on Population?

**DOI:** 10.9745/GHSP-D-24-00121

**Published:** 2024-10-29

**Authors:** Win Brown, Karen Hardee

**Affiliations:** a University of Washington, Department of Global Health; Center for Studies in Demography and Ecology, Seattle, WA, USA.; b Hardee Associates, Arlington, VA, USA.

## Abstract

The International Conference on Population and Development Programme of Action and its Cairo Consensus can help ensure that policy responses uphold human rights and gender equality, thereby serving as the singular global reference that could bring opposing voices in the population debate together.

## Key Messages

- The International Conference on Population and Development (ICPD) has come to represent shorthand for the shift from family planning programs funded based on macrodemographic variables to focus global attention on sexual and reproductive health and rights and on the lives and opportunities of individual women.
- This shorthand ignores language in the ICPD Programme of Action and the Cairo Consensus that was forged, acknowledging that enhancing individual health and rights would contribute to the demographic transition.
- Country policies since ICPD have expanded to incorporate components of the Cairo Consensus, focusing on population and sustainable development, with an expanded focus on reproductive health, individual choice, and women’s empowerment. Yet, the global discourse on “population” has grown more toxic.
- As we near the final years of the Sustainable Development Goal Agenda, we argue that the continued polarization of views about the role of population in addressing the world’s most urgent global health and public policy issues can be bridged by referring back to the comprehensive, inclusive, and progressive ICPD Programme of Action.

## INTRODUCTION

In 1798, Thomas Malthus famously theorized that population growth would always outpace food supply, resulting in human misery unless strict controls on reproduction were introduced. Since Malthus,^1^ debates about the role of population dynamics in socioeconomic development policies and programs have been lively and contentious.^2^^–^^5^ Feminist academics and advocates have raised important concerns about the extent to which population policies that include demographic goals are susceptible to coercion and violation of human rights in programming. As a result, the inclusion of population dynamics in the global development discourse is viewed as problematic,^6^^–^^10^ with suspicion leveled at the terms family planning (FP)^11^ and “voluntary” FP.^12^ The critique has been influential^13^; a consequence has been to effectively remove population dynamics from the discourse on addressing some of today’s most pressing issues.^14^

As we commemorate 30 years since the 1994 International Conference on Population and Development (ICPD) in Cairo, the so-called “toxification of the population discourse”^15^ is salient in the global development community.^14^^,^^16^^,^^17^ Although debates about the role of population in policies and programs predate ICPD,^18^ at Cairo, the field made its fundamental shift. With wide participation of civil society in the 3 successive preparatory consultations that provided evidence leading up to the conference, feminist advocates succeeded in steering the ICPD Programme of Action to a new orientation of reproductive health (RH) and reproductive rights (with the full term sexual reproductive health and rights [SRHR] coined after ICPD) within an expanded focus on population and development.^19^^,^^20^ The reorientation is widely viewed as the paradigm shift in the population and development field, providing a blueprint for a new era of policies and programs.

The rights-oriented paradigm shift^21^ has resulted in a degree of partisanship about the role of population dynamics in global development. Although the conference included a focus expanded from “population” to “population and development,” May noted that discussions of population were delegitimized after Cairo, and “the challenge will be to reconcile the macro-demographic approach with human rights considerations.”^22^ At issue is the extent to which demography and attention to population are compatible with approaches to policies and programs that prioritize individual rights. In this article, we refer to demography as the scientific study of human population focused on the roles of fertility, mortality, and migration in affecting the size, composition, distribution, and age structure of populations. Population dynamics broadly refers to the use of demographic methods to describe and predict how these components change over time; population growth is subsumed under population dynamics and specifically denotes the rate (typically the annual rate) at which a population increases. Our use of “population” varies by context but refers generally to the terms described here and how they may or may not be emphasized in policy and program discourse.

For some constituencies, the fulfillment of individual reproductive health and rights is considered to be incompatible with and be a separate pursuit from broader development, sustainability, and demographic considerations.^14^

Thirty years after ICPD, the world population has grown from 4.6 billion to 8.2 billion.^23^ Two-thirds of the world’s countries have fertility rates that are below what demographers call replacement level, while a smaller number of countries have high fertility rates and are growing rapidly. For example, nearly 20% of all countries and areas, including China, Italy, the Republic of Korea, and Spain, are experiencing so-called “ultra-low” fertility, where the average woman has fewer than 1.4 live births over her lifetime. By contrast, over 10% of countries and areas—mostly in sub-Saharan Africa—have fertility levels of 4 births or more per woman.

In this context of a demographic divide, discussions about the effects of population dynamics on socioeconomic development are no longer confined to concerns about rapid population growth. Now, with population dynamics underlying so many of the world’s priority development issues and with constituencies polarized over whether and how to treat population as a term, the question is whether the ICPD Programme of Action provides an answer.

We say yes. The role of population in affecting issues of human, societal, and sustainable development was canonized in the ICPD Programme of Action, along with much-needed and long-neglected attention to human rights and women’s empowerment. However, despite this, ICPD is frequently referred to as the signature event that once and for all de-linked demography from FP and RH. For many, particularly at the global level, referencing ICPD is shorthand for the shift away from justifying FP programs on the basis of economic and demographic concerns about population growth, which were viewed as associated with population control and human rights abuses. If this is the prevailing view, then “population” and “demography” become trigger words, inhibiting the discourse. We say that the discourse on population can be normalized by taking a closer look at how the participants at Cairo shaped the final Programme of Action.

The role of population in affecting issues of human, societal, and sustainable development was canonized in the ICPD Programme of Action.

As noted by Ashford^24^:

*A turning point in international discussions on population was the 1994 International Conference on Population and Development (ICPD), held in Cairo. Whereas earlier world conferences on population had focused on controlling population growth in developing countries, mainly through family planning, the Cairo conference enlarged the scope of policy discussions. Governments now agreed that population policies should address social development beyond family planning, especially the advancement of women, and that family planning should be provided as part of a broader package of reproductive health care. Underlying this new emphasis was a belief that enhancing individual health and rights would ultimately lower fertility and slow population growth.*

Fred Sai, a co-chair of the ICPD, among other prominent roles,^25^ wrote that, in addition to focusing on women’s empowerment and SRHR, “Cairo signaled an understanding that population is at least seen as part of the necessary investment in people, without which none of our development or environmental problems will be solved.”^26^ Yet, 30 years after the historic consensus achieved at Cairo, there is continued aversion to the term population and continued avoidance of “population” in global discourse and development agendas. In the context of this discourse, we examine the effects of ICPD on national development policies, noting that the implementation of these policies is beyond the scope of this commentary. As the field commemorates ICPD, we explore whether the Cairo Consensus (Box 1^27^^,^^28^) and the ICPD Programme of Action are fit for purpose in guiding policies inclusive of both rights and population dynamics, both for countries with growing populations and for those with shrinking populations.

BOX 1 The Cairo Consensus

- Facilitate the demographic transition
- Provide voluntary family planning in the context of reproductive health
- Improve maternal and child health outcomes
- Promote empowerment of women
- Protect individual human rights
- Ensure broad participation in policy development, notably women and youth

## THE EVOLUTION OF POPULATION POLICIES

Strategies to address issues of population dynamics related to socioeconomic development have been debated for decades,^4^^,^^29^ with discussions of population dating back to Aristotle and Plato.^30^^,^^31^ Based on concerns about and fears of possible detrimental effects of rapid population growth on socioeconomic development, the use of population policies as instruments to address population dynamics gained traction in the 1970s,^18^ with differing perspectives on what the focus on the population policies should be.

The slogan best remembered from the 1974 United Nations (UN) World Population Conference (WPC)—the first of 3 international population conferences convened under the auspices of the UN—was that “development is the best contraceptive,” with representatives from developing countries proclaiming that economic development would take care of population growth. The World Population Plan of Action from the 1974 WPC urged countries “to consider adopting population policies within the framework of socio-economic development, which are consistent with human rights and national goals and values.”^19^ The human right to FP had been articulated at the 1968 International Human Rights Conference that proclaimed (para. 16) the basic right of parents “to determine freely and responsibly the number and the spacing of their children.”^32^

Ten years after the 1974 WPC, at the 1984 International Population Conference in Mexico City, which came at a time of increasing concern in developing countries about rapid population growth, the U.S. delegation posited that, on balance, population growth had a neutral effect on economic development. This was widely viewed as an unpopular outlier position that, according to Basu, “led to a flurry of academic and non-academic activity to dispute this opinion, and yielded a vast body of new work on the consequences of population growth.”^33^

By the 1994 ICPD, a number of studies had been conducted to measure the association between population growth and economic development, with varying results and conclusions.^34^^,^^35^ At the same time, the right to FP that had been established in 1968 was reaffirmed in 1974, 1984, and 1994 at ICPD. At Cairo, human rights came to the fore in debates about population and the appropriate roles of policies and programs.

## DEBATES ABOUT POPULATION AT THE INTERNATIONAL CONFERENCE ON POPULATION AND DEVELOPMENT AND FORGING THE CAIRO CONSENSUS

Contention about “population” came to a head in Cairo,^36^^–^^38^ where it was less about whether it was important or not, but that the field’s focus on population, and particularly on lowering fertility, was impinging on human rights, particularly on women’s rights. Critics faulted governments and donors for promoting FP as an easier technological fix rather than tackling the range of social and economic factors associated with underdevelopment and poverty.^39^ Writing just weeks before the ICPD, the prominent economist and philosopher Amartya Sen called for delegations and participants to avoid partisanship on the issue of population^2^:

*Few issues today are as divisive as what is called the “world population problem.” With the approach this autumn of the International Conference on Population and Development in Cairo…these divisions among experts are receiving enormous attention and generating considerable heat. There is a danger that in the confrontation between apocalyptic pessimism, on the one hand, and dismissive smugness, on the other, a genuine understanding of the nature of the population problem may be lost…. If the propensity to foresee impending disaster from overpopulation is strong in some circles, so is the tendency, in others, to dismiss all worries about population size.*

Although RH, based on the World Health Organization’s definition at the time, along with rights, was elevated at ICPD, the Programme of Action includes 16 chapters, only 1 of which focuses on reproductive rights and reproductive health (Box 2).^41^ Many chapters include the word population and related components, including growth and structure, mortality and morbidity, and migration (both internal and international). The Programme of Action also includes chapters on gender equality, equity, and women’s empowerment and on education. The first thematic chapter focuses on interrelationships between population, sustained economic growth, and sustainable development, which includes environmental sustainability. Paragraph 1.12 of the Programme of Action stated^41^:

BOX 2 International Conference on Population and Development Programme of Action Chapters

- Preamble
- Principles
- Interrelationships between population, sustained economic growth and sustainable development
- Gender equality, equity, and empowerment of women
- The family, its roles, rights, composition, and structure
- Population growth and structure
- Reproductive Rights and Reproductive Health
  - Reproductive rights and reproductive health
  - Family planning
  - Sexually transmitted diseases and prevention of human immunodeficiency virus (HIV)
  - Human sexuality and gender relations
  - Adolescents
- Health, Morbidity and Mortality
- Population Distribution, Urbanization, and Internal Migration
- International Migration
- Population, Development and Education
- Technology, Research and Development
- National Action
- International Cooperation
- Partnership with the Non-Governmental Sector
- Follow-up to the Conference

*The present Programme of Action recommends to the international community important population and development objectives, as well as qualitative and quantitative goals that are mutually supportive and of critical importance to these objectives. Among these objectives and goals are: sustained economic growth in the context of sustainable development; education, especially for girls; gender equity and equality; infant, child and maternal mortality reduction; and the provision of universal access to reproductive health services, including family planning and sexual health.*

The Programme of Action was shaped by the Cairo Consensus that emerged at ICPD among diverse participants, including country delegations from the North and South, population experts and social development advocates, feminists, and “neo-Malthusians,” whose perspective addresses the relationship between population, economic growth, and environment resources.^27^^,^^28^^,^^42^ Hodgson and Watkins described the shaky common ground reached in Cairo as one in which^37^:

*Neo-Malthusians commit themselves to a gender equity strategy for attaining population stabilization, and programmatically agree to supplement family planning activities with reproductive health activities that add several times to program costs. Feminists thereby gain an ally for gender equity campaigns and a commitment for additional funding for women’s health programs. They offer, in turn, only lukewarm support for neo-Malthusian goals, and that support is heavily circumscribed with human rights rhetoric regarding choice.*

DeJong explained that “the consensus that emerged at Cairo was arrived at through a complicated inter-weaving of interests, movements and intellectual trends, as well as owing much to the particular nature of politics both global and national at the time.”^43^

These diverse groups forged a consensus that resulted in the Programme of Action and that provided a blueprint for policies and programs. According to the Cairo Consensus (Box 1), countries should facilitate the demographic transition, a theory regarding 5 stages of human populations moving from high mortality and high fertility to low mortality and low fertility^40^ (e.g., see Objective 6.3 of the Programme of Action).^41^ In the Cairo Consensus, countries should also provide voluntary FP in the context of RH care, improve maternal and child health outcomes, promote empowerment of women, and protect individual human rights. Furthermore, the development of policies should include broad participation of a range of stakeholders, most notably women and youth.

Diverse groups forged a consensus at ICPD that resulted in the Programme of Action and provided a blueprint for policies and programs.

In line with the Cairo Consensus, the ICPD Programme of Action did not contain population targets but instead a 20-year plan with^41^:

*… important population and development objectives, as well as qualitative and quantitative goals that are mutually supportive and of critical importance to these objectives. Among these objectives and goals are: economic growth in the context of sustainable development; education, especially for girls; gender equity and equality; infant, child and maternal mortality reduction; and the provision of universal access to reproductive health services, including family planning and sexual health” (Para. 1.12).*

Repudiating “population control,” the ICPD Programme of Action clarified in Para. 7.12 that “demographic goals, while legitimately the subject of government development strategies, should not be imposed on FP providers in the form of targets or quotas for the recruitment of clients.”^19^

## POST-CAIRO INTERPRETATION OF THE INTERNATIONAL CONFERENCE ON POPULATION AND DEVELOPMENT

The Cairo Consensus emerged over 9 days and nights of deliberations. It represented a holistic, comprehensive approach to population and development policy, allowing countries to optimize achievement of development goals by integrating population dynamics and RH and ensuring the rights of individuals and couples to decide the number and spacing of their children. Many groups at the time of ICPD were advocating for such an approach. For example, some women’s health advocates were calling for “feminist population policy” that acknowledged population stabilization as a favorable outcome for ensuring RH and rights and women’s empowerment.^37^^,^^44^^–^^46^ However, despite the pluralism that characterized the Cairo Consensus and the inclusive and integrative language in the ICPD Programme of Action, today, the ICPD Programme of Action is more commonly understood in more partisan terms as the fundamental shift away from population dynamics.

Dixon-Mueller explained that suggestions for refocusing policies “are in no way intended to denigrate the seriousness of the population problem or to suggest that nothing needs to be done.”^44^ As described by Carmen Barroso, then Regional Director of International Planned Parenthood Federation, Western Hemisphere Region and longtime RH and human rights scholar and advocate^47^:

*The [ICPD] conference document presents a new perspective, in which high rates of population growth are understood as an interdependent and aggravating factor – rather than the cause – of problems such as poverty and environmental degradation. Even more critically, it places women’s wellbeing at the centre of population policy and points to human rights of individuals to determine and plan family size.*

Before ICPD, Berer, then editor of the journal *Reproductive Health Matters* (now *Sexual Reproductive Health Matters)*, asked activists to “acknowledge that the world cannot sustain an unlimited number of people”^37^ and urged them to be involved in shaping population policies to meet the needs of women.

In short, the language of conversations and recommendations from 1994 included population and demography, even from the most activist supporters of the new RH paradigm. Today, the language has narrowed, particularly at the global level. The point to be made here is that such narrowness cannot be accurately attributed to ICPD.

## POST-INTERNATIONAL CONFERENCE ON POPULATION AND DEVELOPMENT COUNTRY POPULATION AND DEVELOPMENT POLICIES

How have country population and development policies fared in the 30 years since ICPD? Beginning in 1963, the UN Population Division has been querying countries about a range of population issues and whether their countries were taking any steps to alter the demographic dynamics.^48^ Between 2015 and 2019, “nearly three quarters of the governments of the 197 member and non-member States of the UN had policies to influence fertility levels.”^48^ Among those, 69 countries included policies to lower fertility (35%), 55 to increase fertility (28%), and 19 to maintain current levels of fertility (10%). Fifty-four countries had no official policy on population (27%).

### Influence of the International Conference on Population and Development Programme of Action in Population Policy Documents

Governments develop their population policies to reflect their national context, with growing participation of a range of national and development partner stakeholders.^49^^–^^51^ Early population policies were promoted by technical experts, and the first population policies were adopted with little public discussion.^52^^–^^55^ The ICPD expanded the range of stakeholders, notably civil society and nongovernmental organizations, involved in determining the scope of population policies. For example, in contrast to its first population policy in 1967, Kenya’s 2012 Population Policy for National Development was developed with broad participation. Key to the passage of the policy “…was the patient, inclusive nature of the consultative process … that solicited input from stakeholders from the very beginning.”^50^ Kenya received an award during the World Health Assembly in 2013 for its participatory process of population policy development in 2012; the process to develop its new population policy has been equally participatory with multisectoral and multistakeholder engagement to identify current challenges and policy solutions.^56^

At the same time, countries’ policies have been influenced by international and regional conferences and agreements and notably ICPD.^19^^,^^22^^,^^42^^,^^57^^,^^58^ In a review of 15 post-ICPD national and subnational population and development policies in Africa (11) and Asia (4), all countries focused on lowering fertility, and all were aligned with the components of the Cairo Consensus.^59^ The 15 policies reviewed were focused on population and sustainable development, including environmental sustainability, and all had expanded language on RH and women’s empowerment.

Countries’ population policies have been influenced by international and regional conferences and agreements and notably ICPD.

Thus, ICPD did broaden the scope of population policies to encompass the range of components in the Cairo Consensus. For example, the 1998 Population Policy for South Africa noted that “the ICPD offered a useful new international perspective on population and development issues.”^60^ As explained by Geraldine Fraser-Moleketi, then Minister for Welfare and Population Development, in the foreword to the 1998 Population Policy for South Africa^60^:

*The concerns spelt out in the policy pertain to problems associated with poverty, gender discrimination, environmental degradation, gross socio-economic inequities between rich and poor and between the urban and rural sections of the population, premature mortality, especially in infants, and the threat of HIV/AIDS and other sexually transmitted diseases, teenage pregnancies, the lack of expertise in the population and development field and a general lack of reliable population data and information on population and development interrelationships. Obviously, this policy focuses on more than just fertility trends and fertility control.*

Similarly, Nigeria’s 2021 National Policy on Population for Sustainable Development noted that in addition to being in accord with the country’s constitution and policies, the policy’s guiding principles are derived from the 1994 ICPD Programme of Action and its “unfinished agenda” and are aligned with the 2030 Agenda for Sustainable Development and its 17 SDGs as well as the Agenda 2063 for African Development.^61^

### Respect for Rights

Robinson argued that “population policy adoption after the conference became a way of demonstrating a commitment to human (reproductive) rights.”^58^ She noted that all sub-Saharan African countries signed the ICPD Programme of Action and that the word “rights” was mentioned in post-ICPD policies 8.8 times on average, compared to 6.4 times in policies formulated before ICPD, a statistically significant difference.^58^ In line with the right proclaimed at the 1968 International Human Rights Conference^32^ and upheld in population conferences since, including ICPD, Botswana’s 1997 National Population Policy “upholds the basic rights of couples and individuals to RH and to decide freely and responsibly the number and spacing of their children, and to have access to information and education to make an informed choice; and the means to do so.”^62^

Tanzania’s 2006 National Population Policy also affirms human rights-related principles from ICPD.^63^ Uganda’s 2020 National Population Policy includes 7 principles to guide implementation; of those, 5 focus on human rights, including, among them, the right to RH.^64^ In line with ICPD, Malawi’s 2023 National Population Policy “positions fulfilment of rights especially those of women and girls to be paramount in order to achieve slowed population growth and an inclusively wealthy middle-income nation.”^65^ The Center for Reproductive Rights has highlighted South Africa’s Population Policy for South Africa (1998) as an exemplary policy focused on a rights-based approach.^46^

### Facilitating the Demographic Transition: Bringing Population Dynamics Into Alignment With Development and Focus on Family Planning, Reproductive Health, and Women’s Empowerment

While there has been an expanded focus on reproductive health and rights since ICPD, the rationale for population and development policies has continued to reflect governments’ focus on aligning population dynamics with development, well-being, and resources.^51^^,^^64^^,^^66^^,^^67^ According to remarks made by Dr. Jotham Musinguzi, Director General of Uganda’s National Population Council, in a webinar on the Four Dividends, the 2020 National Population Policy for Uganda focuses on quality of life and well-being, including health, education, and jobs, rather than on population size or growth.

Since its establishment as a country in 1971, Bangladesh has had 3 population policies, which have benefited from strong government support. Its first policy in 1976 aimed to reduce the population growth rate from 3% in 1976 to 2.5% in 1978, with an emphasis on FP and integrating population as an integral part of national development planning.^68^ The Bangladesh Population Policy 2004 retained a focus on reducing population growth to reach a stable population by 2060. At the same time, the 2004 policy was influenced by the 1994 ICPD, with a shift from FP to inclusion of RH.^69^ This focus on well-being is also illustrated in Bangladesh’s most recent policy.^70^ The vision of the Bangladesh Population Policy 2012 is to “develop a healthier, happier and wealthier Bangladesh through planned development and control of the nation’s population.”^66^ Like Bangladesh’s 2004 policy, the 2012 policy includes explicit language recommending client-centered FP and RH care, among other strategies, including addressing population and the environment.

Some countries, particularly in sub-Saharan Africa, are focused on achieving the demographic dividend and have sought to shape their population policies accordingly. The United Nations Population Fund (UNFPA) defines the demographic dividend as “the temporary economic benefit that a country can earn from a significant increase in the ratio of working-age adults relative to young dependents that is created by a rapid decline in birth rates.”^71^ The idea is that as populations shift from high to low fertility rates, the resulting change in age structure could create an economic “dividend” achieved by favorable ratios of the sizes of working-age populations to young and old dependency groups. The demographic dividend came into the economic development literature only 25 years ago, highlighting the role of changing age structures in explaining the economic booms achieved by several South and East Asian countries.^72^ The concept is important because the focus on age structure gave economists and demographers a new way to frame the role of population dynamics in economic development.^73^

Looking beyond FP, the Sindh [Pakistan] Population Policy 2016 noted that achieving the demographic dividend “is possible only through active involvement of all key stakeholders and Departments working on female education, status of women, youth development, economic growth and employment generation, addressing regional inequities, conservative attitudes including male preference and young age at marriage, etc.”^67^ Vietnam has been working to maintain its age structure. In its Population Strategy to 2030 (the latest of several population policy updates since 1991), Vietnam sought to maintain its current favorable age structure to “maximize the advantages of a golden population, creating strong momentum for the country’s rapid and sustainable development.”^74^

### Additional Policy Foci Since Cairo: Addressing the Population-Environment/Climate Change Nexus and Increased Attention to Migration

Although climate change per se was not the prominent global issue in 1994 that it is today, the link between population and the environment was a major topic of discussion at Cairo.^19^ Over the past 25 years, countries have included population-environment linkages in their development policies, with increasing attention to climate change. Ghana’s 1994 National Population Policy recognized the need to address environmental issues in its section on environmental programs, including by “developing and enforcing laws and regulations that protect the environment [and] Ensuring judicious exploitation of the nation’s natural resources.”^75^ Uganda’s National Population Policy 2020 addresses the environment and climate with an objective to strengthen an integrated approach to population, development, and environment,^64^ and climate change adaptation and resilience are among the 6 priority areas in Malawi’s 2023 National Population Policy.^65^

Over the past 25 years, countries have included population-environment linkages in their development policies, with increasing attention to climate change.

As the world has progressively urbanized since ICPD, population policies have increased attention to migration,^19^ which, along with fertility and mortality, comprise the 3 primary factors that explain changes in population size, composition, and distribution. Migration is included in population and sustainable development policies, particularly managing population movement between rural and urban areas and addressing rapid urbanization. For example, noting that the country’s population is concentrated in about 20% of the land area, Kenya’s 2012 Population Policy for National Development highlights “the continued strain on the existing urban infrastructure, particularly on housing, transportation, educational and health facilities, and employment” in urban areas.^76^

Kenya’s policy calls for refocusing migration to small- and medium-sized cities, addressing issues in informal settlements, and ensuring RH services for the urban and rural poor and in other hard-to-reach areas. Mauritania’s 2005 National Statement of Population Policy, an update from its 1995 policy, included 2 objectives related to internal and international migration, including addressing spatial distribution of the population and strengthening the monitoring of the situation of immigrants, welcoming returnees, and monitoring international migration.^77^

## FRAGILE GLOBAL PERCH OF THE FULL INTERNATIONAL CONFERENCE ON POPULATION AND DEVELOPMENT PROGRAMME OF ACTION

While countries have continued paying attention to population dynamics, the global discourse on population remains fraught. Mention of the word “population” connotes a toxic allusion to previous periods in the history of demography and FP, including those that are associated with eugenicist ideologies.^8^^,^^9^^,^^78^ Despite the progressive language of the ICPD Programme of Action, its full agenda has never enjoyed consistently strong support from the development community.^79^ Many of the early studies supporting the hypothesis that rapid population growth was a constraint to economic development have been discounted.^9^^,^^11^ Although ICPD was the first UN conference to present a budget to achieve its recommendations, the Programme of Action has not succeeded in garnering sufficient funding.^14^^,^^80^ Its fragile standing in the development community was reflected in the absence of FP and reproductive health and rights in the original formulation of the Millennium Development Goals (MDGs, 2000-2015), with MDG5b, “achieving universal access to RH” added belatedly.^81^

By the time the development community adopted the Sustainable Development Agenda (2016– 2030), the goals were underpinned by equity and empowerment considerations and arguably more closely resembled the ICPD Programme of Action^29^ SRHR considerations, which were represented in the Sustainable Development Goals (SDGs) as universal access to RH care (an indicator of SDG 3) and the gender equality and women’s and girls’ empowerment indicators of SDG 5.

Yet, at the same time, the UN Population Division’s 2022 World Population Prospects report noted that the “cumulative effect of lower fertility, if maintained over several decades, could be a more substantial reduction of global population growth in the second half of the century.”^82^ In a 2022 UN publication titled “Why Population Growth Matters for Sustainable Development,” Wilmoth and colleagues described the effects of rapid and sustained population growth as “exacerbating and generating social, economic, and environmental challenges that range from food insecurity and gender inequity to environmental degradation.”^83^ From these points of view, high fertility and rapid population growth present challenges to the achievement of sustainable development and magnify the environmental impact of harmful economic processes. Likewise, sustained low and very low fertility also have implications for sustainable development.^84^ UNFPA has reiterated that while policies must be grounded in respect for human rights, population dynamics have an impact on development and thus must be incorporated into policies.

*Population size and structure impact a country’s economy as well as its ability to provide social protections and access to health care, education, housing, sanitation, water, food and energy.* ^85^

Population is conspicuously left out of global discussions and strategies to address climate change,^86^ though population is a variable in climate models^87^ and its contribution to carbon emissions is significant. The 2022 technical summary of the IPCC Sixth Assessment Report of Mitigation of Climate Change Working Group) stated that^88^:

*Globally, gross domestic product (GDP) per capita and population growth remained the strongest drivers of CO2 emissions from fossil fuel combustion in the last decade (robust evidence, high agreement).*

It is important to note that this message gets edited out of summaries for policymakers.^89^

## CONTINUED TENSION AROUND “POPULATION”

Some advocates and academics persist in positioning attention to “population” as code for coercive FP^7^^,^^8^ or arguing that “today’s population debate presents a false set of choices, focusing attention on how to control the world’s population and foreclosing the question about whether doing so would actually solve any of the world’s problems.”^9^ Senderowicz and Valley sum up the continuation of the opposition raised leading up to ICPD^11^:

*Family planning remains an appealing global health intervention to many in this post-ICPD era. Contraceptive programs are portrayed as a cost-effective solution to the haunting specter of overpopulation, and the myriad-purported benefits of family planning dovetail with other progressive goals including women’s health, environmental protection, and poverty alleviation. In their quest to solve so many challenges of sustainable development using contraception, however, many family planning programmers mistakenly frame fertility as a fundamental cause of these challenges, leaving the real culprits at large. These true culprits—entrenched health inequities, overconsumption and waste, unequal distribution of resources, and extractive colonial economic relationships, among others—lack quick, technological solutions. And while many scholars and activists have addressed these complex issues head on, many others have remained fixated on fertility reduction and contraceptive use as a silver bullet. The promise of reproductive rights and autonomy articulated at the ICPD, therefore, remains unfulfilled as these instrumentalist arguments remain so dominant.*

Others counter that there has been insufficient attention to population growth since ICPD, including the need for stronger focus on the effects of population on the environment.^90^^–^^92^

Leading up to the Cairo ICPD, Amartya Sen noted^2^:

*There are reasons to worry about the long-term effects of population growth on the environment; and there are strong reasons for concern about the effects of high birth rates on the quality of life, especially of women.*

May contended that in focusing on individual needs, population programs have “lost sight of the big demographic picture as well as the huge logistical requirements to serve burgeoning populations.”^93^ In its manual for conducting Population Situation Analysis, UNFPA wrote that population trends “have an impact on compliance with rights, either because the location of individuals is an obstacle in terms of their access to services, or because the growth of the population or specific sub-groups generates pressures that are hard to attend, for increased resources for social programmes or for services that affect environmental sustainability.”^94^

Addressing the tension regarding population in 2009, 15 years after the ICPD, a Kenyan leader of a foundation population program was heckled when she stressed at a nongovernmental organization forum that ignoring population jeopardizes achievement of the ICPD goals. She used the example of Kenya to explain that “no one doubts the value of empowering women through education, but when population grows this fast, countries are simply not able to sustain their development. And when education and health systems are overwhelmed or fail all together, I can assure you that it is women and girls who suffer first and most.”^95^

In her book *The Means of Reproduction: Sex, Power and the Future of the World*, Goldberg wrote that^96^:

*To say that poor countries aren’t responsible for resource scarcity…doesn’t change the fact that it is going to make it even more difficult for them to absorb millions of new people…A massive investment in women’s education, birth control access, and income generation would lessen the danger that the world’s population would outstrip the planet’s resources.*

## CALLS TO BUILD BRIDGES BETWEEN THE SEXUAL AND REPRODUCTIVE HEALTH AND RIGHTS AND DEMOGRAPHIC COMMUNITIES

In 2014, Newman and colleagues attempted to form a bridge between the SRHR and demographic communities to engage in population dynamics to ensure inclusion of SRHR in the post-2015 development agenda.

*It is possible to care about population dynamics (including ageing and problems faced by countries with a high proportion of young people) and care about human rights at the same time…if sexual and reproductive health and rights advocates do not participate in the population dynamics discourse, the field will be left free for those for whom respecting and protecting rights may be less of a priority.* ^97^

In 2019, Barroso and Sinding, identifying “population growth and sustainable development, people and the planet, and reproductive health and rights as the 3 most important issues facing the field today,” called for “both/and” solutions.^79^

Ensuring attention to rights is increasingly important. In 2019, Indian Prime Minister Narendra Modi said that a large population was obstructing India’s development.

*We have to think if we can do justice to the aspirations of our children. There is a need to have greater discussion and awareness on population explosion.* ^98^

Ensuring attention to rights is also increasingly important in the context of nationalist ideologies and in countries facing very low fertility and population growth rates,^84^ with the possibility of rolling back access to RH services and compromising human rights in the name of increasing fertility through pronatalism.^99^^–^^101^ The elevated focus on rights in the Programme of Action has meant that people can use human rights standards and principles and national and global human rights commissions to hold their governments accountable for rights violations.^102^

Broadening the discussion beyond RH, more than 11,000 scientists worldwide have endorsed 6 steps to reduce the effects of climate change. Among the steps is stabilizing population growth within a framework that ensures social integrity and upholds human rights.^103^ Analysis of nearly 100 options for reducing carbon emissions by Project Drawdown, an initiative that advances effective, science-based climate solutions and strategies, identified FP and education as among the top 10 solutions. They stressed that^104^:

*Rights-based, voluntary family planning and universal, high-quality education are essential human rights. They generate numerous direct benefits for gender equality, improved health and well-being, economic development, and more. Slower global population growth, a cascading outcome of increased family planning and rising education levels, contributes to reduced greenhouse gas emissions. … It is critical to center human rights, full bodily autonomy, and gender equality, and recognize that benefits to the planet are positive ripple effects of access and agency.*

These attempts at détente have been mostly rebuffed, and the emergence of climate change and environmental degradation as policy issues have come with renewed calls to silence talk about population.^105^^–^^107^ Galavotti characterizes arguments such as Project Drawdown’s “noxious.”^108^ In 2021, the International Planned Parenthood Federation wrote^109^:

*Different stakeholders have pointed to contraception as an important intervention for climate change mitigation. Project Drawdown…includes FP alongside girls’ education among the top 10 of its climate solutions….Rhetoric and actions suggesting curbs on the fertility of women and girls as a solution for social and environmental ills have a long and dangerous history and still manifest today.*

Many other organizations, including Women Deliver,^110^ the Women and Gender Coalition, and the SRHR and Climate Justice Coalition,^111^ have made similar statements. The SRHR and Climate Justice Coalition has provided members with messaging guides to counter the “unhelpful and possibly dangerous” population control narrative,^107^ seeking to close off any mention of population in discussions of climate change. In response, some members of a Population Health and Environment policy and practice group have countered that^112^:

*When people everywhere can exercise bodily autonomy about whether and with whom to have sex, exert control over their fertility through the realization of universal access to SRHR, and ensure all births are planned, the end result of slower population growth can contribute to a long-term reduction in global emissions through global demographic shifts. Disparaging contraception and family planning run counter to achieving universal access to SRHR.*

Still, in many global convenings, any statements conveying the idea that population growth in and of itself could be deleterious to development are quickly condemned as egregious—as out-of-step Malthusian thinking with undertones of coercion and racism.^113^^,^^114^

The loudest critical voices tend to be from the Global North, with the perspectives from other regions, such as sub-Saharan Africa, underrepresented in “global discussions of population dynamics and in international environmental governance.”^113^ For example, during the UN Climate Change Conference in 2021, Nancy Tembu, Malawi’s Minister of Forestry and Natural Resources, stressed that “addressing gender issues and population growth needed to be at the center of climate mitigation and adaptation.” Her comments contrasted with the rest of the summit’s focus, which barely addressed population dynamics.^113^ With sub-Saharan Africa projected to become the most populous region in the second half of the century, with high vulnerability to the effects of climate change and environmental degradation, hearing more voices and perspectives from this region on this topic at the global policy level is critical.^115^

Also seeking to bridge the divide, the Population Institute, in 2024 “Revitalizing Population and Development in the 21st Century,” observed that the Programme of Action^116^:

*Laid out an early framework for a rights-based approach to assessing and addressing intersecting challenges related to population and development that have come into even sharper focus in the 21st century. Thirty years on, this framework has only gained meaning and value in a diverse world population now grown to 8 billion people, facing immense challenges related to governance, security, food security, environmental sustainability, and human rights.*

## DISCUSSION

Taken as a whole, the ICPD Programme of Action represents the single most important reference guiding work in the field of population and development. To this day, it represents the signature consensus among country delegations to inform population and development policies and programs, and its progressive language not only set the stage for the SDGs but also was remarkably forward-looking in its final orientation. Writing about ICPD in 2009, 15 years after the landmark conference, Roseman and Reichenbach wrote that the strength of the Programme of Action, and thus the Cairo Consensus, is that^117^:

*The conceptual underpinnings of ICPD can be—and have successfully been—incorporated into arguments regarding public health, human rights, demographics, development, and empowerment, all of which are relevant to broader health and development debates.*

The declaration from the 57th UN Commission on Population and Development in 2024 on their review of the ICPD Programme of Action at 30 recognized that population dynamics, including changing age structures, growth rates, urbanization, and migration, will continue to shape the world and future generations and calls for the full, effective and accelerated implementation of the Programme of Action.^118^ The Commission on Population and Development recognizes that the ICPD Programme of Action contains language that is very current today, 30 years later. For example, the Programme of Action recognized women in poor countries as the rightful owners of reproductive agency and that they should be empowered to have control over their own bodies.

The Cairo Consensus is relevant for countries facing low fertility with concerns about population aging and the effects of population decline on socioeconomic development. Any policies in those countries also need to respect rights and promote women’s empowerment—principles established with global agreement nearly 30 years ago at ICPD in Cairo.

In this commentary, we have attempted to show that many countries remain concerned about population growth rates and have responded with policies focused on ensuring that population trends are in line with development and available resources, including through expanding access to voluntary FP as part of RH, along with interventions to change social and gender norms and empower women. We have attempted to show that if countries choose to have a continued focus on population dynamics, then their population policies can be grounded in the human rights of individuals and couples to make decisions—freely and responsibly—on the number and spacing of their children, a right established more than 50 years ago.^32^

We have attempted to show that many countries remain concerned about population growth rates and have responded with policies focused on ensuring that population trends are in line with development and available resources.

We have shown how countries have embraced the Cairo Consensus and how it remains problematic for some others at the global level. We have argued that a narrow focus on SRHR is a misappropriation of the Cairo Consensus, but we acknowledge that its resiliency is nuanced. Outside of the content of various countries’ population policies, we show that population has been intentionally marginalized from the development discourse. Thirty years after the ICPD, while the Cairo Consensus has been of greater service to the field than the various parties in 1994 anticipated, the reality is that the Consensus has proven resilient in some ways (e.g., country policies) but not in others (e.g., the global discourse).

With respect to resiliency at the country level, we have suggested that the ICPD Programme of Action provided countries with a sanctioned reference and set of guidelines for policymakers to implement programs that today, with no irony, we would characterize as people-centered development, with expanded focus on RH, individual choice, and women’s empowerment. Although the Cairo Consensus stands as the authoritative standard for policy decades after it was forged at ICPD, it’s true that the ICPD Programme of Action has failed to galvanize the full set of global development constituencies engaged in the field of FP and SRHR, one result of which we claim is the so-called toxification of the population discourse. As we near the final years of the SDG Agenda, we contend that the continued polarization of views about the role of population in addressing the world’s most urgent global health and public policy issues could be bridged by referring back to the comprehensive, inclusive, and progressive ICPD Programme of Action.

Countries—including those now facing fertility rates below replacement level—are likely to continue with broad attention to population issues, including effects on socioeconomic development. The ICPD Programme of Action and its Cairo Consensus help to ensure that policy responses are scrutinized regarding upholding human rights and gender equality, thereby serving as the singular global reference that could bring opposing voices in the population debate together.

## Notes

**Peer Reviewed**

**First Published Online:** October 1, 2024.
